# Effect of tracheostomy on pulmonary mechanics: An observational study

**DOI:** 10.4103/1658-354X.62606

**Published:** 2010

**Authors:** Khalid Sofi, Tariq Wani

**Affiliations:** *Staff Physician, Department of Anesthesia, King Abdul-Aziz Medical City, King Fahad National Guard Hospital, Riyadh, Columbia, Missouri, USA*; 1 *Assistant Professor, Department of Anesthesia and Perioperative Medicine, University of Missouri, Columbia, Missouri, USA*

**Keywords:** *Tracheostomy*, *weaning*, *mechanical ventilation*

## Abstract

**Background:**

This study was undertaken to find out the effect of early tracheostomy on weaning from mechanical ventilation. Pulmonary mechanics and arterial blood gases were assessed before and after tracheostomy in patients with severe head injury (Glasgow coma score < 8) requiring prolonged mechanical ventilation.

**Patients and Methods:**

The study included 20 mechanically ventilated patients of either sex between 20 and 45 years of age, who had suffered brain injury due to head trauma during admission (Glasgow coma scores of < 8). Mean airway pressure, peak airway pressure, plateau pressure, PaO_2_ and PaCO_2_ were measured 24 h before and after tracheostomy. Static and dynamic compliances were calculated.

**Results:**

Plateau airway pressures were not affected by tracheostomy, but peak airway pressure was reduced (29.90 ± 3.21 cm H_2_O before tracheostomy versus 24.30 ± 1.83 cm H_2_O after tracheostomy, *P* < 0.001). Dynamic compliance, but not static compliance, was improved by tracheostomy. Tracheostomy did not affect PaCO_2_, but it improved PaO_2_ (83.09 ± 5.99 mmHg before versus 90.84 ± 5.61 mmHg after, *P* < 0.001).

**Conclusions:**

The work of breathing through a tracheostomy tube may be less than through an endotracheal tube of same internal diameter.

## INTRODUCTION

Tracheostomy is a surgical procedure routinely done in critically ill patients requiring prolonged mechanical ventilatory support. Tracheostomy might facilitate weaning by reducing dead space and airway resistance, and by improving secretion clearance. This reduces the likelihood of tube obstruction by inspissated mucus, makes the patient more comfortable, requiring less sedation and reducing the likelihood of aspiration through improved glottic function.[1] Endotracheal tubes (ETTs) increase dead space and elevate airway resistance, which could lead to excessive ventilatory support. Tracheostomy might determine whether a patient is ventilator dependent or successfully weaned.[2] There is generally an improvement in clinical status following tracheostomy. The exact mechanism for improved clinical status remains ill-defined.

Breathing humidified air through a tracheostomy tube differs from normal breathing via the intact upper airway primarily with respect to the volume of dead space and the impact of airway resistance. These factors are usually cited when explaining why a difficult to wean patient might be able to breathe unassisted through a tracheostomy tube but not through a translaryngeal ETT or, by inference, following extubation. It has been demonstrated that the *in vivo* resistance of ETTs exceeds the *in vitro* resistance because of thermal liability of materials and the tortuous translaryngeal path.[3] In patients projected to need more than 14 days of ventilatory support, percutaneous dilatational tracheostomy performed in the first two days rather than after day 14, greatly reduced the number of ventilator and intensive care unit days, and decreased both the incidence of pneumonia and overall mortality.[4]

A major obstacle in studying the effects of tracheostomy on weaning is the inability to predict which patients will require prolonged ventilatory support. Impaired consciousness may not by itself be a reliable predictor of the need for prolonged airway protection, yet this is one the most commonly invoked justifications for tracheostomy.[5]

The aim of this study was to assess pulmonary mechanics and arterial blood gases before and after tracheostomy in patients with severe head injury (Glasgow coma score < 8) requiring prolonged mechanical ventilation.

## PATIENTS AND METHODS

### Patients

This study was conducted after approval of the Ethical Research Committee at Sher-i-Kashmir Institute of Medical Sciences. The study included 20 adult patients aged 20–45 years of either sex who had suffered head trauma due to vehicular accidents and fall from height. All patients included in this study had on admission Glasgow Coma Scores of < 8 and their lungs were mechanically ventilated. Informed consent was obtained from the immediate family members. We considered the need for prolonged mechanical ventilation for further care as the indication for surgical tracheostomy. Patients with fever or subcutaneous emphysema were excluded from the study.

### Methods

Tracheostomy was planned on day 5 of ventilation. ETT was replaced by tracheostomy tube of the same internal diameter. All patients′ lungs were ventilated with Puritan Bennett Ventilator (series 7200). Patients who exhibited bleeding from the tracheostomy wound were excluded from the study. We recorded mean airway pressure, peak airway pressure, plateau pressure, PaO_2_ and PaCO_2_ 24 h before the planned tracheostomy and 24 h after tracheostomy.

Static and dynamic compliances were calculated from the obtained measurements. Dynamic compliance was calculated by the equation:

$\mathrm{Dynamic}\;\mathrm{compliance}\;(\mathrm{mL}/\mathrm{cm}\;H_{2}O)=\frac{\mathrm{Tidal}\;\mathrm{volume}}{\mathrm{Peak}\;\mathrm{airway}\;\mathrm{pressure}-\mathrm{PEEP}}$

Static compliance was calculated by the equation:

$\mathrm{Static}\;\mathrm{compliance}\;(\mathrm{mL}/\mathrm{cm}\;H_{2}O)=\frac{\mathrm{Tidal}\;\mathrm{volume}}{\mathrm{Plateau}\;\mathrm{airway}\;\mathrm{pressure}-\mathrm{PEEP}}$

The ventilatory settings and modes were maintained during the course of measurement. Tidal volume was set at 6–8 mL/kg.

### Statistics

Data were analyzed using student′s *t*-test. A P value of less than 0.05 was considered statistically significant. Data are presented as the mean ± SD.

## RESULTS

Patient demographic data and diagnosis are shown in Table 1. Mean airway pressure and plateau pressure were not affected by tracheostomy [Table 2]. Tracheostomy did however significantly reduce peak airway pressure: (29.90 ± 3.21 cm H_2_O before tracheostomy versus 24.30 ± 1.83 cm H_2_O after tracheostomy) *P* < 0.001.

**Table 1:** Demographic data of patients

No.	Sex	Age (yrs)	Diagnosis	Mode	Flow (L/min)	PEEP (cm H_2_O)	FiO_2_(%)
1	M	24	Multiple contusions with EDH left frontoparietal area	A/C	55	0	45
2	M	28	Acute SDH	A/C	50	3	50
3	M	32	Acute SDH	A/C	60	3	55
4	M	23	Acute SDH with EDH	A/C	60	3	40
5	F	29	EDH with intraventricular bleed	A/C	45	5
6	F	26	Diffuse axonal injury	A/C	50	5	45
7	M	21	Diffuse axonal injury	A/C	50	5	55
8	M	42	Multiple contusions with EDH left temporal area	A/C	50	4	70
9	F	37	EDH with intraventricular bleed	A/C	50	4	40
10	M	31	Acute SDH with EDH	A/C	65	4	45
11	F	26	Multiple contusions with EDH right frontoparietal area	A/C	50	4	40
12	M	25	Acute SDH with # base of skull	A/C	55	4	40
13	F	34	Multiple contusions with EDH left temporal area	A/C	50	4	50
14	M	28	Acute SDH with EDH	A/C	55	4	50
15	M	30	EDH with intraventricular bleed	A/C	55		40
16	F	36	Acute EDH with # base of skull.	A/C	50	4	60
17	F	44	EDH with intraventricular bleed	A/C	45		40
18	F	23	Multiple contusions with EDH left temporal area	A/C	45	4	55
19	M	37	Acute SDH with EDH	A/C	60	5	45
20	M	33	EDH with intraventricular bleed	A/C	50	0	45

Static compliance (*C*_stat_) improved after tracheostomy, but this change was statistically insignificant, as plateau pressure was not affected. In contrast, tracheostomy improved dynamic compliance (*C*_dyn_) by a significant amount; because of decrease in peak airway pressure (20.90 ± 2.78 before tracheostomy and 27.32 ± 3.36 after tracheostomy) *P* < 0.001.

PaCO_2_ values before and after tracheostomy were similar [Table 2]. PaO_2_ values, however, were significantly improved after tracheostomy (83.09 ± 5.99 mmHg before versus 90.84 ± 5.61 mmHg after tracheostomy) *P* < 0.001.

**Table 2:** Lung mechanics before and after tracheostomy

	Pre-tracheostomy	Post-tracheostomy	*P* value
Mean airway pressure cm H_2_O	3.49 ± 1.22	3.24 ± 1.23	0.060
Peak airway pressure cm H_2_O	29.90 ± 3.21	24.30 ± 1.83	0.001
Plateau airway pressure cm H_2_O	19.05 ± 4.16	18.40 ± 3.10	0.800
Dynamic compliance ml/cm H_2_O	20.90 ± 2.78	27.32 ± 3.36	0.001
Static compliance ml/cm H_2_O	38.30 ± 7.04	40.32 ± 6.98	0.100
PaO_2_ mmHg	83.09 ± 5.99	90.84 ± 5.61	0.001
PaC_2_ mmHg	31.82 ± 2.40	32.36 ± 2.00	0.500

Data are presented as mean ± SD.

## DISCUSSION

Head trauma patients constitute about 50% of all neurosurgical patients to our ICU. Majority of the patients admitted to ICU have low GCS scores and require prolonged mechanical ventilation.

In our institution, we perform tracheostomy early in the ICU stay of these patients based on the consensus opinion that early tracheostomy will provide better pulmonary toilet, a shorter ICU stay and quicker weaning from the ventilator.

We studied the effects of tracheostomy on resistive and elastic components of lung dynamics and its effect on blood gases. The resistive component of lung dynamics is described by peak airway pressure and dynamic compliance; the elastic component is described by plateau pressure and static compliance. Our study showed improved peak airway pressure and, hence, dynamic compliance, which confirms the consensus opinion that the decreased length of tracheostomy tube, compared with ETT, decreases the resistance to breathing. This change was associated with improved PaO_2_ in patients with tracheostomy.

Most published discussion of the impact of tracheostomy have been based on the results of studies with surrogate end points such as its effects on dead space or work of breathing, rather than on data of actual weaning from ventilatory support. If weaning and extubation are lumped together, then an unsuccessful attempt to extubate may mean reintubation, which is inconvenient, uncomfortable and potentially dangerous for the patient. On the other hand, if the spontaneous breathing trial is unsuccessful in a patient with tracheostomy, the artificial airway remains in place and resuming ventilatory support is quick and easy. While it seems clear that there are differences between tracheostomy and ETTs with respect to dead space and resistance, for most patients these differences are unlikely to explain the ready conversion to spontaneous breathing that often occurs after tracheostomy.

Rumbak *et al*.[4] reported a multicenter clinical study of early versus delayed tracheostomy in 120 medical ICU patients, who were considered to need prolonged mechanical ventilation. These researchers found that early tracheostomy reduced mortality by half and pneumonia by three-fourths. In addition, there was, on average, a 10-day reduction in the need for ventilatory support in the patients receiving early tracheostomy.

Davis *et al*.[6] suggested that decreased airway resistance and intrinsic positive end expiratory pressure (PEEPi) play a partial role in freeing the patient from mechanical ventilation after tracheostomy. The clinical benefits of these improved physiological variables have yet to be elucidated.[7] Lin *et al*.[8] found that patients experienced very little improvement in pulmonary mechanics and only a small improvement in peak inspiratory pressures after tracheostomy. The authors proposed that patients with better underlying lung mechanics had a better chance of weaning from mechanical ventilation after tracheostomy. The work of breathing is reduced after tracheostomy, compared with breathing through an ETT[6] possibly because the tracheostomy tube offers less resistance than does the thermo liable ETT, which can become deformed in the upper airway. Successful discontinuation of mechanical ventilation after tracheostomy represents improved airway control compared with spontaneous breathing via the intact upper airway in select patients.

The ability to wean a patient from mechanical ventilation after tracheostomy frequently occurs after failed extubation attempts. In these patients, tracheostomy allows improved secretion clearance, allows simple initiation and discontinuation of ventilatory support and eliminates the variable of upper airway control.

Like our study, Lin *et al*.[8] also found that tracheostomy reduced the peak inspiratory pressure significantly and that measurement of pulmonary mechanics before tracheostomy could be useful for predicting the weaning from mechanical ventilation. The decrease in airway peak inspiratory pressure through the tracheostomy may be a result of the decrease in dead space ventilation and partially due to a decrease in patient discomfort by tracheostomy tube compared with ETT.

A major obstacle to studying the effects of tracheostomy on weaning is the inability to predict which patients will require prolonged ventilatory support. While such prediction may be relatively straightforward in some conditions (such as bulbar weakness or high cervical spinal cord injury), it is woefully inaccurate in the absence of these conditions. Impaired consciousness may not by itself be a reliable predictor of the need for prolonged airway protection, yet this is one of the most commonly invoked justifications for tracheostomy.[3] Another barrier to the performance of good clinical studies is the variability in approach to weaning from ventilatory support among different clinicians and different institutions. The other variables that would affect the performance of any study seeking to clarify the role of tracheostomy in weaning are who performs the procedure and how experienced they are, in performing it.

Tracheostomy does reduce dead space, in comparison with the non-intubated state; however, the difference between an ETT and a tracheostomy tube with respect to dead space is small.

We conclude, therefore, that the work of breathing through a tracheostomy tube may be less than through an ETT of the same internal diameter.

## References

[1] Pierson DJ (2005). Tracheostomy and weaning. Respir Care.

[2] MacIntyre NR (2004). Evidence-based ventilator weaning and discontinuation. Respir Care.

[3] Wright PE, Marini JJ, Bernard GR (1989). *In vitro* versus *in vivo* comparison of endotracheal tube airflow resistance. Am Rev Respir Dis.

[4] Rumbak MJ, Newton M, Truncale T, Schwartz SW, Adams JW, Hazard PB (2004). A prospective, randomized, study comparing early percutaneous dilational tracheotomy to prolonged translaryngeal intubation (delayed tracheotomy) in critically ill medical patients. Crit Care Med.

[5] Coplin WM, Pierson DJ, Cooley KD, Newell DW, Rubenfeld GD (2000). Implications of extubation delay in brain-injured patients meeting standard weaning criteria. Am J Respir Crit Care Med.

[6] Davis K, Campbell RS, Johannigman JA, Valente JF, Branson RD (1999). Changes in respiratory mechanics after tracheostomy. Arch Surg.

[7] Epstein SK (2005). Anatomy and physiology of tracheostomy. Respir Care.

[8] Lin MC, Huang CC, Yang CT, Tsai YH, Tsao TC (1999). Pulmonary mechanics in patients with prolonged mechanical ventilation requiring tracheostomy. Anaesth Intensive Care.

